# Empowerment Among Adolescent Girls in Nepal: A Concept Mapping Exploratory Study

**DOI:** 10.9745/GHSP-D-23-00010

**Published:** 2024-06-27

**Authors:** Jessica G. Burke, Sara Baumann, Jennifer Jones, Niva Joshi, Pema Lhaki

**Affiliations:** a Department of Behavioral and Community Health Sciences, University of Pittsburgh School of Public Health, Pittsburgh, PA, USA.; b Department of Epidemiology, University of Pittsburgh School of Public Health, Pittsburgh, PA, USA.; c Nepal Fertility Care Center, Sanepa, Lalitpur, Nepal.

## Abstract

**Background:**

The concept of empowerment is challenging to operationalize and measure; it is multidimensional, the outcomes are not always directly observable, and meanings of empowerment are highly contextual and socially and culturally situated. This study aimed to explore perspectives of empowerment among adolescent girls in Nepal to identify statements for inclusion in a context-specific empowerment measure.

**Methods:**

We used a participatory and mixed method research method called concept mapping in 3 districts in Nepal. Three sequential concept mapping sessions were used to solicit, organize, and process how participants responded to the prompt: “The life of an adolescent girl improves when she has/can…” The Concept Systems Global software was used to manage and analyze the concept mapping brainstorming, sorting, and rating data using established tools, such as multidimensional scaling and cluster analysis.

**Results:**

Concept mapping was conducted with 113 participants, including national experts, program staff, adolescents, and their mothers. They identified 105 items that fall into a 4-cluster solution: education and knowledge, decision-making, supports and skills, and physical infrastructure. Rating data uncovered there was some overlap between the top 10 most important items between the national-level experts and other stakeholder groups; however, several components associated with empowerment differed by stakeholder group in terms of importance.

**Conclusion:**

This research represents a critical step in exploring definitions of empowerment among adolescent girls in the Nepal context and with expert input. These results led to the development of a contextually specific definition of empowerment. Researchers and practitioners interested in developing context-specific understandings of complex topics that incorporate community voices and perspectives could use a similar concept mapping approach in other countries to explore various topics with diverse populations.

## Abstract (precis)

This study used concept mapping, a community-engaged and participatory research method, to identify a wide range of factors that help “define” a context-specific concept of empowerment among adolescent girls in Nepal that will form the foundation for developing a Nepal-specific empowerment measurement for program evaluation.

## Teaser

**Key Findings:**

- In an effort to develop a contextually specific empowerment measurement tool for adolescents in Nepal, 4 stakeholder groups at the national, community, and village levels were engaged in concept mapping activities to provide insights into the components of empowerment.
- Empowerment among adolescent girls in Nepal is influenced by a wide range of factors, including components of education and knowledge, decision-making, social supports and skills, and physical infrastructure.
- Items rated the most important were “sex education” for adolescents, “hygiene” for mothers, “education” for community-level staff, and “treat and be treated equally” for national-level experts, illustrating the distinct interpretations of empowerment and highlighting the need for incorporating a wide range of voices to define empowerment comprehensively.

**Key Implication:**

- The concept of empowerment should be explored and conceptualized in the specific context in which it is being used to help develop more effective approaches to measuring the impact of empowerment interventions.

## INTRODUCTION

First presented in the early 1980s,^1^ the focus on empowerment as an important development strategy for improving the lives of adolescent girls has grown exponentially over the past decade.^2^^,^^3^ Despite increased attention and intervention efforts, the specific dimensions of empowerment are elusive and challenging to enhance and measure because the concept is multidimensional and the outcomes are not always directly observable.^4^ Rowlands questioned empowerment definitions and underscored why it is important to better understand the concept^5^:

*Unless empowerment is given a more concrete meaning, it can be ignored, or used to obscure, confuse or divert debates. The failure to define and explore the practical details of how empowerment can be achieved considerably weakens the value of the concept as a tool for analysis or as part of a strategy for change.*

Empowerment is widely recognized as a complex concept and is often considered both a process and an outcome with multiple dimensions. Rappapon et al. suggest that it is easier to define the absence of empowerment by focusing on powerlessness and helplessness than it is to operationalize the positive and growth-oriented constructs of empowerment.^6^ Recent work by Cattaneo and Goodman^7^ defined empowerment as “a meaningful shift in the experience of power attained through interactions in the social world.” Other scholars focused on how empowerment relates to an individual’s ability to control her own life and make independent decisions while failing to address the factors that facilitate and constrain an individual’s agency and action.^8^ As noted by Narayan, empowerment is a relational concept that is influenced by multiple levels of factors. According to Pratley’s literature review,^9^ the 5 conceptual dimensions of empowerment include psychological, social, economic, legal, and political components. Underpinning these definitions are 3 levels of influence: individual agency, relationships, and structural environments.

While there are likely components of empowerment that apply universally, empowerment manifests differently across different populations and settings. As a result of this recognition, there is growing consensus that measures of empowerment should be conceptualized within specific populations and settings rather than at a global level or with universal measures.^7^^,^^10^^–^^12^ What constitutes empowerment for 1 group of individuals in a specific context might not hold true for another group in a different context. Therefore, it’s crucial to tailor measures of empowerment to the specific population and setting being studied.

In Nepal, empowerment interventions are increasingly being used to address the numerous challenges that adolescent girls encounter, including the lack of awareness and knowledge about sexual and reproductive health,^13^ restricted access to sexual and reproductive health services and information, menstrual restrictions,^14^ child early and forced marriages/unions,^15^ adolescent pregnancy,^16^ poor nutrition,^17^ school dropout, unsafe abortion, sexually transmitted infections, HIV/AIDS, and substance abuse.^16^ This is particularly critical in Nepal, where 37% of girls marry before age 18 years and 10% are married by age 15 years.^18^ According to Nepal Demographic Health Survey 2022 data, 14% of adolescent women aged 15–19 years have ever been pregnant.^19^ Though the government of Nepal has recognized adolescents and youth as an underserved and vulnerable population with specific sexual and reproductive health needs and has developed the National Adolescent Sexual and Reproductive Health Programme,^20^ effective approaches for measuring the impact of such interventions on youth empowerment are urgently needed.

In Nepal, empowerment interventions are increasingly being used to address the numerous challenges that adolescent girls encounter, including the lack of awareness and knowledge about sexual and reproductive health.

This study intentionally built on an existing program in Nepal, the Rupantaran program, which focuses on empowering adolescent girls to explore perspectives of empowerment among adolescent girls and used a 2-phased approach to develop a contextually specific empowerment measurement tool. In this article, we present the Phase 1 exploratory research concept mapping process and results. Specifics about how the concept mapping results were used in a case/control study paired with factor analysis (Phase 2) to develop the Power in Nepali Girls empowerment scale are published elsewhere.^21^

## METHODS

The Rupantaran program, implemented by the Nepal Fertility Care Center (NFCC), a local nongovernmental organization, is a holistic social and financial skills program designed to empower out-of-school adolescent girls with the knowledge and skills needed to make important life decisions, such as enhanced literacy and numeracy skills. NFCC has been implementing the Rupantaran program in Saptari, Rautahat, and Dhanusha districts since 2014 with financial support from UNICEF Nepal.

The Rupantaran program, implemented under UNICEF’s education unit in Nepal, consists of informational sessions conducted 3 times a week by social mobilizers and facilitators with out-of-school adolescent girls. The 15-module program aims to empower adolescent girls to participate in decisions affecting their lives and become change agents in their communities. The sessions include information on a range of topics, including self-realization, rights and responsibilities, nutrition, financial planning, sexual and reproductive health, and violence. The Rupantaran training also provides information on health and hygiene and entrepreneurship for improving livelihoods. A total of 15 parent sessions are held concurrently alongside the adolescent modules.

This project was conducted in partnership between practitioners at NFCC and researchers at the University of Pittsburgh (PITT), with funding from UNICEF Regional Office for South Asia. Through the established partnership, the team was able to leverage practitioner expertise of longstanding expertise implementing the Rupantaran program, paired with researcher expertise in community-engaged research and concept mapping to explore how empowerment is defined, with the ultimate goal of developing a concept-specific measure for the Nepal context. Concept mapping is a participatory, structured method that is uniquely suited for developing group consensus about complex topics and illustrating relationships between concepts. The method has been successfully used to inform the development of frameworks and inform measurement development.^22^^,^^23^ The method provides quantitative structure and objectivity to qualitative data and is an efficient way to identify group consensus.^24^

For this project, concept mapping was conducted in the Rupantaran program districts at the time of the study, specifically Saptari, Dhanusha, Rautahat (located in Province 2 along the Southern border with India), as well as the capital, Kathmandu. These districts were selected by the Nepali government to receive the Rupantaran program because of the region’s high primary education dropout rates among adolescent girls and the significant gender equity issues, including child early and forced marriages/unions and early pregnancy.

A purposive sample of 113 individuals representing 4 stakeholder groups participated in concept mapping, specifically national-level experts (n=7), community-level program staff (NFCC and UNICEF) (n=14), adolescent Rupantaran program participants from 3 villages (n=48), and mothers of the Rupantaran program participants (n=44). We intentionally included 4 stakeholder groups based on anecdotal findings and our hypothesis that these 4 groups have slightly different conceptualizations of the concept. This study design allowed us to capture those similarities and differences and contributed to the development of a comprehensive, Nepal-specific, and community-informed definition of empowerment.

We intentionally included 4 stakeholder groups based on our hypothesis that these groups have slightly different conceptualizations of the concept.

The team recruited 4 groups of participants who have been actively engaged with the Rupantaran program and as a result well positioned to provide the needed insights about components of empowerment. National-level experts and community-level program staff participants were identified by NFCC through their existing relationships. National-level experts included representatives from the Department of Women & Children and the National Women Commission of the Nepal Government, UNICEF, Restless Development, and NFCC. Community-level participants included staff from UNICEF Janakpur, Life Nepal, and NFCC. The adolescent Rupantaran program participants and their mothers were recruited based on the recommendation of NFCC community-level staff familiar with the participants and parents. Consistent with the exploratory and qualitative nature of this study, we recruited participants who have been actively engaged with the program and, as a result, well positioned to provide the needed insights about components of empowerment. Participants gave their written consent. Parental permission and youth assent were obtained for adolescents aged younger than 18 years.

### Data Collection

The PITT team provided a 3-day concept mapping methods training to NFCC team members and 8 facilitators, who are fluent in both Nepali and English. This training was conducted via interactive videos, Skype calls, engaging presentations, and in-person practice exercises in Nepal. After completing the training, the local Nepali facilitators conducted 3 concept mapping sessions, separated by key stakeholder group to ensure participants felt comfortable sharing their ideas (i.e., brainstorming, sorting/rating, and interpretation). The same participants were invited to all 3 sessions, and the average group size for each session was 14 participants. Each session depended on information gathered in prior sessions. The session 1 brainstorming activity included a total of 113 participants. For session 2, sorting and rating, 111 participants returned, in which 100% completed the rating activities and 47% completed the sorting activity. For session 3, interpretation, 109 of the original 113 participants attended the discussions.

#### Session 1: Brainstorming

During session 1, participants were asked to brainstorm responses to the focal prompt, “The life of an adolescent girl improves when she has/can…” To guide responses, the facilitators provided a brief description of the project’s interest in empowerment. Participants were asked to take a couple of minutes to think about their responses and then to share them with the group. They were also given the opportunity to write anything that they did not feel comfortable sharing publicly. Multiple team members were involved in the process of consolidating the responses into a single list of “items” to be used in subsequent concept mapping activities. That process involved removing duplicates, combining items with similar meaning, and ensuring that the participants’ language and original meaning were maintained. Each of the items was then printed onto notecards; 1 item per card for the session 2 sorting activity.

#### Session 2: Sorting and Rating

During session 2, participants sorted all the items into piles based on perceived similarity and placed them in baskets provided by the study team. The number of piles/baskets was determined by each participant based on their perceptions of similarities between the 105 items. After sorting the items, the participants then named each pile. Participants also ranked each item in order of importance and clarity on a 5-point Likert scale. The importance scale asked: How important is each of the items for supporting increased capacity and power of girls? The clarity scale asked: How clear is each item (are you able to easily understand the meaning of the item)?

#### Session 3: Interpretation

In session 3, facilitators shared importance rating results from session 2 with the participants to initiate a qualitative data interpretation session. The facilitator sought group input about how each of the 29 items aids in empowerment to improve the lives of girls. In addition, participants were asked about how each item should be revised to improve clarity and understanding and to center on adolescent girls. Those 29 items served as the foundation for the case/control survey conducted as part of Phase 2.^21^

### Data Analysis

In preparation for data analysis, all data collected during the 3 sessions were translated from Nepali to English by the NFCC team and cross-checked by 2 Nepali-speaking team members of the PITT team. Excel spreadsheets and Concept Systems Global, a web-based platform, were used to manage and analyze the brainstorming, sorting, and rating data (https://conceptsystems.com/home) and the results were shared between the Nepal and U.S.-based research team members using a secure online Box folder. This article provides details about the complete concept mapping dataset, including the items and concepts, and explores rating differences by stakeholder type.

Established concept mapping procedures, including non-metric multidimensional scaling, which uses square total similarity matrices, were applied to the sorting data from completed datasets to create spatial point maps on an X-Y graph, with the relative distance between the items representing perceived similarities and differences. The stress test value for this project was 0.33, indicating a good overall fit and that the distances on the point map were consistent with the values in the input similarity matrix. Most concept mapping projects have stress values between 0.205 and 0.365 (mean of 0.285). Using hierarchical cluster analysis, based on the minimization of the sum of squares of the distances between all items, the team then created cluster maps illustrating group consensus regarding item categories. Bridging values for each item within the cluster were used to identify the anchors of the cluster. Bridging values reflect the likelihood that participants sorted statements similarly. The anchor items and the cluster titles provided by participants were used to generate the final cluster labels. Using the rating data categorized by each stakeholder group, the top 10 most important items for adolescent girls’ empowerment for each group were identified.

### Ethical Approval

This study was reviewed and approved by the Nepal Health Research Council (748-2019) and the PITT Institutional Review Board (STUDY119060250).

## RESULTS

### Participant Characteristics

Phase 1 participants had diverse educational backgrounds; 100% of the national-level experts and 77% of the community-level staff had above a high school education; 100% of the mothers had no education and 100% of the adolescents had below a high school education (appropriate given their age). The average age of the mothers was 44 years, a bit higher than that of the national-level experts (38 years) and the community-level staff (29 years). The average age of the adolescent participants was 13 years for all 3 study districts.

### Components of Empowerment Among Nepali Adolescent Girls

After combining the brainstorming data from all participants (collected through 8 brainstorming sessions) and removing the duplicates, 105 unique factors were identified in response to the focal prompt: “The life of an adolescent girl improves when she has/can…” These factors ranged widely and included items such as “knowledge about child early and forced marriages/unions and child rights” (item 32), “capacity to negotiate” (item 3), “good sanitation” (item 96), and “community support” (item 65).

The Figure presents the results of the multidimensional scaling and the cluster analysis and provides insight into the perceived similarities between the items. We explored cluster solutions ranging from 2 to 20 clusters to assess the item groupings. The 4-cluster solution illustrated fairly distinct concepts: (1) education and knowledge, (2) decision-making, (3) supports and skills, and (4) physical infrastructure. Table 1 lists the item content of the 4 cluster domains. The number listed next to the item statement can be used to locate the item on the cluster map (Figure). The points on the map are on an X-Y graph with the relative distance between the points (items) representing perceived similarities and differences. For example, item 3, “capacity to negotiate,” is located in the bottom right corner of the map and was perceived to be quite different from item 96, “good sanitation,” which is in the top left corner of the map. The cluster boundaries illustrate group consensus regarding item categories, in which items closer together are perceived to be similar, and items farther away are perceived to be distinct. Items in the education and knowledge and decision-making clusters were strongly anchored, meaning that most of the participants considered items within each of those 2 clusters to be quite similar and sorted those items together. Items in the physical infrastructure and supports and skills clusters contained a slightly higher bridging score, indicating that there was less consensus about the placement of those items.

**TABLE 1.** List of Empowerment Component Statements Generated from Concept Mapping Sessions, by Cluster

Item	Item Statements
**Cluster 1: Education and knowledge**	
2	Understanding of social and cultural values and life skills
5	Knowledge about the dowry system
16	Knowledge about basic health management
21	Knowledge about safety
27	Knowledge about the age of adolescence
32	Knowledge about child marriage and child rights
34	Information about how to register a marriage certificate
37	Information about birth certificates
42	Information about menstruation
47	Literacy
57	Knowledge about HIV/AIDS
58	Knowledge about first aid
66	Education (including age-specific education)
77	Knowledge about topics such as trafficking of girls
78	Understands the importance of education
89	Sex education
92	Knowledge about how to save money
93	Knowledge about gender and gender roles
101	Knowledge about adolescent friendly government services
102	Knowledge about good habits
**Cluster 2: Decision-making**	
1	Same respect as sons/boys
3	Capacity to negotiate
6	Family that supports her decision making
7	Restricted because of gender roles
8	Supportive parents
10	No discrimination
11	Avoid conflict with other girls
13	Career guidance
14	Studies what is interesting to her
17	Given responsibility regardless of gender roles
20	No discrimination during menstruation
23	Unity and is self-dependent
24	Self-awareness
25	Recognizes her own ability
26	Leadership
28	Make decisions for her family
38	Makes own decisions
39	Equal rights
40	Parents who are empowered and counseled about the right age for marriage of their children
41	Participate in decision about and decide her own marriage
55	Face negativity
59	Express herself
60	Few children
62	Identify opportunities
**Cluster 3: Supports and skills**	
4	Job with fair wages (including factories, agriculture business, opportunities abroad)
9	Improved choices
12	Skills to identify resources
19	Support from friends
22	Control over resources
35	Independently earn and spend money
36	Works per her own interests
43	Participates in decision making
44	Global and universal socialization
45	Be self sufficient
48	Family support for her growth and development
49	Treat and be treated equally
50	Withstand peer pressure
51	New opportunities within and beyond her community
52	Feel safe in the community
56	Life skills
61	Valued by family
65	Community support
70	Social support
74	Progressive guidance and support from family and others
75	Her ideas and decisions heard and respected
80	Share her beliefs
85	Free to express herself
87	Security and justice
88	Child and adolescent friendly rules, regulations, laws, and rights at provincial and local government levels
90	Skills training (including tailoring, beauty parlor)
91	Attend community meetings
94	Positive and supportive environment
97	Choose
99	Supports and is helped by other girls
**Cluster 4: Physical infrastructure**	
15	Sanitary pads during menstruation
18	Bridge
29	Knowledge about menstruation
30	Transportation
31	Computers
33	Public toilets
46	Clothing for girls
53	Information
54	Immunizations
63	Pitched roads
71	Hygiene
76	Nutrition
79	Water
82	Schools
86	Be neat and clean
95	Supplies (including books, pens)
96	Good sanitation
98	Health resources (including doctors, health posts, hospitals, ambulance services)
100	A supportive environment for child development (nutrition, growth monitoring, checkups, stimulation, sanitation)
105	Markets

**FIGURE:**
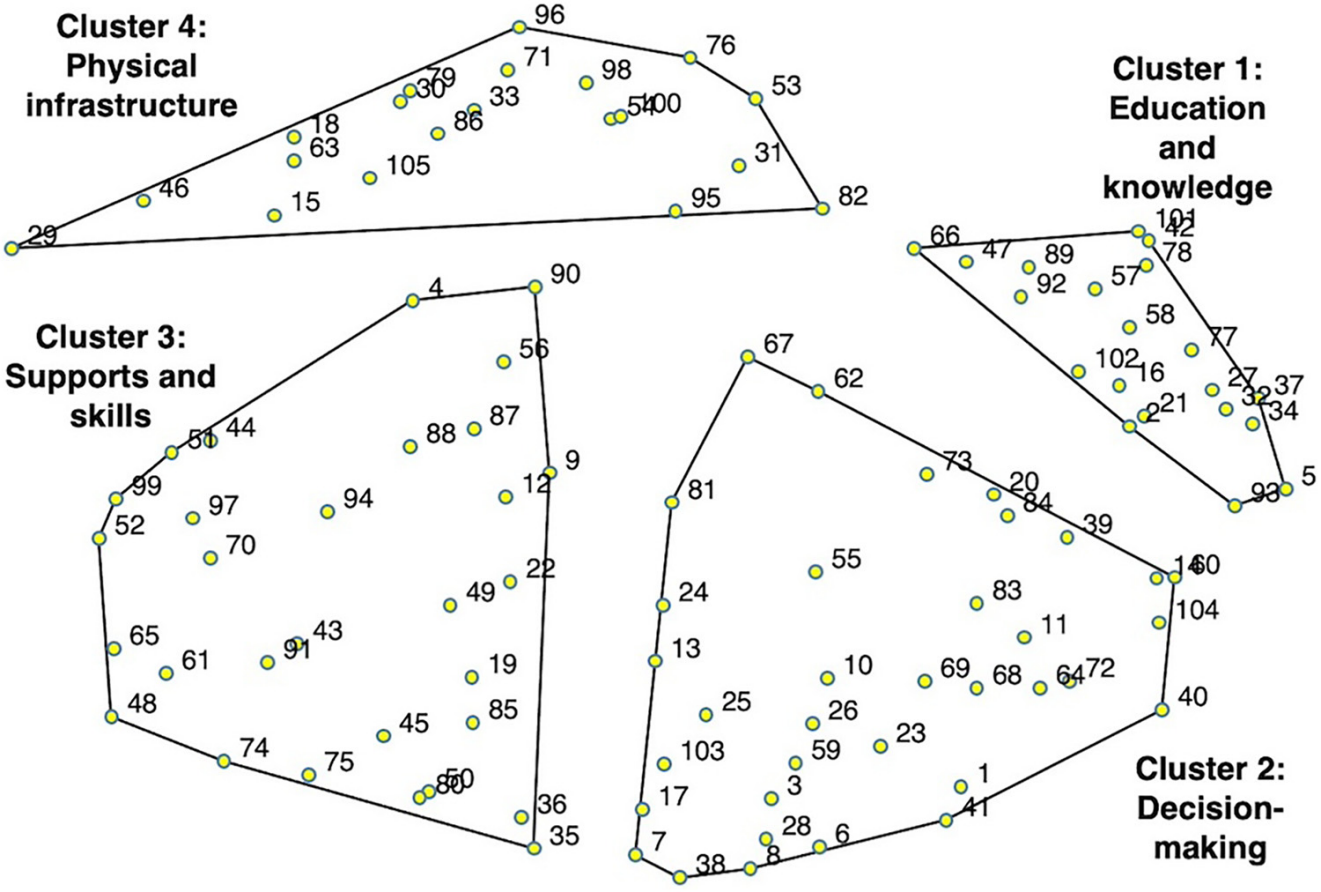
Final Concept Map of Four Clusters of 105 Empowerment Component Items

The final cluster labels were developed based on a review of the items within a cluster and the list of pile names provided by participants. Cluster 1, education and knowledge, includes items such as “knowledge about the dowry system” (item 5) and “knowledge about HIV/AIDS” (item 57). Cluster 2, decision-making, includes items such as “same respect as sons/boys” (item 1) and “capacity to negotiate” (item 3). Cluster 3, supports and skills, includes items such as “being self-sufficient” (item 45) and “social support” (item 70). Cluster 4, physical infrastructure, includes items such as “public toilets” (item 33) and “good sanitation” (item 96).

These 4 clusters provide a valuable “shorthand” for referencing the types of items generated in response to the focal prompt. However, given that the primary focus of the overall project was developing a measurement scale and identifying statements for inclusion in the Phase 2 component, instead of pursing additional exploration of the clusters, we turned our focus to exploring the rating data and exploring the perspectives of the various stakeholder groups.

These 4 clusters provide a valuable “shorthand” for referencing the types of items generated in response to the focal prompt, “The life of an adolescent girl improves when she has/can…”

### Different and Overlapping Perspectives of Empowerment Components

Rating data from the 111 participants who completed the activity revealed which items were believed to be most important for improving the life of an adolescent girl. Because the study design intentionally included multiple stakeholder types and sought wide input into developing a deeper understanding of empowerment in this context, we explored the rating data by stakeholder groups. We discovered there was some overlap between the top 10 most important items between the national-level experts and other stakeholder groups. However, there were several components associated with empowerment that differed by stakeholder group in terms of importance. This signifies that there are distinct interpretations of empowerment at the national level and those in the community and at the village level, highlighting the need for incorporating a range of voices to define empowerment comprehensively. Table 2 presents the top 10 most important items by stakeholder group (a total of 29 items) and the revised statements reflecting suggestions from the participants to improve clarity and understanding. The items rated the most important for improving the life of an adolescent girl in Nepal were “sex education” for adolescents, “hygiene” for mothers, “education” for community-level staff, and “treat and be treated equally” for national-level experts. Item 1, “same respect as sons/boys,” was in the top 10 list of most important items for the national level experts but was not in the top 10 list of most important items for the other 3 stakeholder groups.

**TABLE 2.** Top Ten Most Important Empowerment Components by Stakeholder Group

Item	Original Item	Revised Item Centered on Adolescent Girls	National Experts	Community Staff	Mothers	Adolescents
1	Same respect as sons/boys	My parents treat me the same as sons/boys.	X			
2	Understanding of social and cultural values and life skills	I have basic life skills to take care of myself and/or my family (such as sewing, agriculture, etc.)	X			
3	Capacity to negotiate	I am able to freely share and negotiate my ideas.	X			
4	Job with fair wages (including factories, agriculture business, opportunities abroad)	I am able to be paid the same wages as a male for doing the same work.	X			
6	Family that supports her decision making^a^	My family supports my decisions.	X	X		
8	Supportive parents	My parents support me and help me move ahead with my education.	X			
10	No discrimination^a^	I am not discriminated against on the basis of gender, religion, or caste/ethnicity,	X		X	
17	Given responsibility regardless of gender roles	I am given the same work as boys/sons.		X		
29	Knowledge about menstruation^a^	I am knowledgeable about menstruation.			X	X
30	Transportation	I have access to transportation and a way to travel from one place to another.			X	
32	Knowledge about child marriage and child rights	I am knowledgeable about the harm associated with child marriage and importance of child rights.		X		
38	Makes own decisions	I have the ability to make my own decisions about my life.		X		
43	Participates in decision-making	I participate and provide input into decisions made by my parents.		X		
47	Literacy	I am able to read, write and conduct simple calculations for use in day-to-day life.			X	
48	Family support for her growth and development	My family supports my holistic growth and development.	X			
49	Treat and be treated equally	I treat everyone equally, with respect, and do not discriminate based on age, gender, caste/ethnicity, or religion.	X^b^			
57	Knowledge about HIV/AIDS	I am knowledgeable about HIV/AIDS.				X
58	Knowledge about first aid	I am knowledgeable about first aid.			X	
66	Education (including age-specific education)^a^	I have at least a primary education.		X^b^	X	
71	Hygiene^a^	I maintain my personal hygiene.		X	X	X
73	No discrimination during menstruation^a^	I do not experience discrimination during menstruation.		X		X
75	Her ideas and decisions heard and respected	My ideas and decisions are heard and respected by my parents.	X			
76	Nutrition^a^	I eat nutritious food.		X	X^b^	X
78	Understands the importance of education	I understand the importance of education.				X
82	Schools	I have access to school facilities.				X
89	Sex education	I am educated about sex.				X^b^
96	Good sanitation^a^	I live in a clean and hygienic environment with good sanitation.			X	X
98	Health resources (including doctors, health posts, hospitals, and ambulance services)	I have access to health resources including doctors, health posts, hospitals, and ambulance services.				X
104	Positive thinking	I think positively and have a good attitude.			X	

^a^ Items that appeared in the top 10 for multiple stakeholder groups.

^b^ Rated as most important for that group.

Eight of the 29 items appeared in the top 10 for multiple stakeholder groups, signaling some overlaps in priorities (Table 1). For example, “education” was noted in the top 10 for both community-level staff and mothers at the village level. “Hygiene” was in the top 10 for community-level staff, mothers, and adolescent girls. Mothers and daughters both prioritized “knowledge about menstruation” as a critical aspect of empowerment for adolescent girls.

The interpretation discussion of the 29 items led to the refined statements listed in Table 2 and contributed some insights into the relationship between the items and how each contributes to improving the life of an adolescent girl in Nepal. For example, adolescents talked about how sex education was important because it helps adolescents to learn about the effects of child/early and forced marriages/unions on reproductive health and physical development, and mothers emphasized the importance of hygiene for disease prevention and the health of adolescent girls. Community-level staff discussed how if every adolescent receives a minimum primary education, they might be interested in additional education. National-level experts shared their thoughts about how being treated equally will result in respect and access to education.

## DISCUSSION

This study identified a wide range of factors important for improving the life of an adolescent Nepali girl. According to the study participants, empowerment among adolescent girls in Nepal includes issues ranging from knowledge to decision-making to skills development and structural issues. The inclusion of both decision-making and skills development components is relatively unique within conceptualization and measures of empowerment. For example, the Global Early Adolescent Study scale addresses decision-making agency and resources but does not address knowledge and skills. The Psychological Empowerment Scale includes critical skills and knowledge but does not address decision-making within the context of family and society,^25^ which is likely the case because it is focused on empowerment among parents. These differences underscore the importance of developing a context-specific understanding of empowerment and suggest that existing measurement tools are not as relevant to the cultural context and the populations (e.g., adolescents vs. adults).

According to the study participants, empowerment among adolescent girls in Nepal includes issues ranging from knowledge to decision-making to skills development and structural issues.

Concept mapping is a rigorous method for establishing content validity, supporting researcher decisions, and capturing population perspectives.^26^ Concept mapping is also a unique participatory research method with several strengths that pair well with community-engaged research.^22^ The fact that it employs a series of steps that use different data collection strategies within a structured process was particularly useful for this project and its focus on unpacking the complex topic of empowerment. Despite these strengths and the use of quantitative analytic tools, the selection of the final number of clusters within a concept mapping project is a rather subjective process. In this project, we elected to use a 4-cluster solution to broadly group the items while retaining a focus on the entire set of responses to the brainstorming prompt. Doing so provided structure and adhered to the 6-step concept mapping process without narrowing our focus. The use of the concept mapping method ensures a high degree of confidence that the results are relevant to the intended population and capture multiple aspects of the construct of empowerment among adolescents in the Nepali context. The fact that we documented some distinctions in interpretations of empowerment at the national level versus those in the community and in the village further supports the approach of engaging directly with the community of interest, which, in this case, is adolescents themselves. The incorporation of input from multiple stakeholder types helped avoid the creation of biased findings influenced by a single perspective and supports the external validity and applicability of these findings to the conceptualization of empowerment among adolescent girls in Nepal.

### Limitations

This study has a few limitations worth noting. A primary limitation is that by focusing on stakeholders connected to an existing empowerment program, the results are likely biased by participants’ experiences with the program. Future research should seek input from adolescents and adults not engaged with such programming to explore potential similarities and differences. The non-probability sample techniques and relatively low participation rate for the sorting activity (just under 50%) may limit the generalizability of the sorting results. In addition, there were challenges associated with conducting the study in a remote, rural setting and in a language other than that of the U.S.-based investigators with expertise in concept mapping. However, because of the close partnership with the local Nepali investigators and the inclusion of multiple stakeholder groups within the research, we feel confident that the results accurately capture the complexities associated with empowerment among adolescents in Nepal. The interpretation sessions with participants were limited to primarily clarifying items for use in a future empowerment scale. Therefore, additional qualitative data are still needed that explore the rationale behind each of these items and how they contribute to Nepali youth empowerment.

## CONCLUSIONS

This research represents a critical initial step in defining empowerment among adolescent girls in the Nepal context. These results led to the development of the contextually specific Power in Nepali Girls empowerment scale.^21^ Researchers and practitioners interested in developing context-specific understandings of complex topics that incorporate community voices and perspectives could use a similar concept mapping approach in other countries and populations on a range of health topics.

## Notes

**Peer Reviewed**

**First Published Online:** June 3, 2024
